# The Health-Promoting Potential of *Salix* spp. Bark Polar Extracts: Key Insights on Phenolic Composition and In Vitro Bioactivity and Biocompatibility

**DOI:** 10.3390/antiox8120609

**Published:** 2019-11-30

**Authors:** Patrícia A. B. Ramos, Catarina Moreirinha, Sara Silva, Eduardo M. Costa, Mariana Veiga, Ezequiel Coscueta, Sónia A. O. Santos, Adelaide Almeida, M. Manuela Pintado, Carmen S. R. Freire, Artur M. S. Silva, Armando J. D. Silvestre

**Affiliations:** 1 CICECO—Aveiro Institute of Materials, Department of Chemistry, University of Aveiro, 3810-193 Aveiro, Portugal; patriciaaramos@ua.pt (P.A.B.R.); catarina.fm@ua.pt (C.M.); santos.sonia@ua.pt (S.A.O.S.); cfreire@ua.pt (C.S.R.F.); artur.silva@ua.pt (A.M.S.S.); 2 QOPNA & LAQV-REQUIMTE, Department of Chemistry, University of Aveiro, 3810-193 Aveiro, Portugal; 3 CBQF—Centro de Biotecnologia e Química Fina—Laboratório Associado, Escola Superior de Biotecnologia, Universidade Católica Portuguesa, Rua de Diogo Botelho 1327, 4169-005 Porto, Portugal; snsilva@porto.ucp.pt (S.S.); emcosta@porto.ucp.pt (E.M.C.); mveiga@porto.ucp.pt (M.V.); ecoscueta@gmail.com (E.C.); mpintado@porto.ucp.pt (M.M.P.); 4 Biology Department and CESAM—Centre for Environmental and Marine Studies, University of Aveiro, 3810-193 Aveiro, Portugal; aalmeida@ua.pt

**Keywords:** *Salix* spp. bark polar extracts, phenolic compounds, antioxidant activity, anti-hypertensive potential, antibacterial effect, bioeconomy-based value chain

## Abstract

*Salix* spp. have been exploited for energy generation, along with folk medicine use of bark extracts for antipyretic and analgesic benefits. Bark phenolic components, rather than salicin, have demonstrated interesting bioactivities, which may ensure the sustainable bioprospection of *Salix* bark. Therefore, this study highlights the detailed phenolic characterization, as well as the in vitro antioxidant, anti-hypertensive, *Staphylococcus aureus* growth inhibitory effects, and biocompatibility of *Salix atrocinerea* Brot., *Salix fragilis* L., and *Salix viminalis* L. bark polar extracts. Fifteen phenolic compounds were characterized by ultra-high-performance liquid chromatography-ultraviolet detection-mass spectrometry analysis, from which two flavan-3-ols, an acetophenone, five flavanones, and a flavonol were detected, for the first time, as their bark components. *Salix* bark extracts demonstrated strong free radical scavenging activity (5.58–23.62 µg mL^−1^ IC_50_ range), effective inhibition on angiotensin-I converting enzyme (58–84%), and *S. aureus* bactericidal action at 1250–2500 µg mL^−1^ (6–8 log CFU mL^−1^ reduction range). All tested *Salix* bark extracts did not show cytotoxic potential against Caco-2 cells, as well as *S. atrocinerea* Brot. and *S. fragilis* L. extracts at 625 and 1250 µg mL^−1^ against HaCaT and L929 cells. These valuable findings can pave innovative and safer food, nutraceutical, and/or cosmetic applications of *Salix* bark phenolic-containing fractions.

## 1. Introduction

Presently, the population’s growing rate, the climate change, and the ecosystem degradation have aroused society’s awareness and political decisions for the utmost importance to consume and produce chemicals, energy, and materials in a more ecological and sustainable way. The European Commission launched in 2012 the bioeconomy strategy for addressing the conversion of biomass into bioenergy, food and feed ingredients, fine chemicals, and biomaterials, in order to boost the modernization of economic primary and secondary activities, contributing to reduce fossil fuel dependency and respecting the ecological world’s boundaries [1]. In this context, biorefinery-based industrial plants are attracting broad interest, but the biomass demand has risen the attention to the potential stress on agricultural land use, environment, and ecosystem [2]. In addition to the by-products and wastes of the agriculture, forestry, and food industries, energy crops and short-rotation woody crops can be valuable biomass sources for biorefinery-based plants. *Salix* (Salicaceae), commonly known as willow, is among the most promising short-rotation woody crops, since it grows quickly and can provide high commercial biomass yields, generally reaching 8–10 dry t ha^−1^ year^−1^ in European countries [3]. Additionally, it can be cultivated in abandoned soils, and not necessarily in agricultural fertile fields, leading to a positive impact on biodiversity and rural income [3,4].

Willow has traditionally been used in basket manufacturing and for ornamental aspects, and more recently, for thermal and electricity generation [3]. Furthermore, *Salix* spp. bark extracts are well-known in folk medicine, since the ancient Egyptian, Greek, and Roman civilizations, owing to their analgesic and antipyretic actions which are mainly ascribed to the physiological oxidation of salicin to salicylic acid [5]. In fact, salicin-standardized extracts of *Salix fragilis* L., *Salix purpurea* L., and *Salix daphnoides* Vill. barks are also recommended for lower back pain [6]. Moreover, *Salix* spp. bark polar extracts and phenolic-enriched fractions have exhibited anti-inflammatory, antioxidant, and tumor antiproliferative effects, which have been related with the presence of catechin and procyanidins, instead of the extracts’ marker component salicin [7,8,9]. Other phenolic compounds, namely, acetophenones (e.g., picein), chalcones (e.g., isosalipurposide), and flavanones (e.g., naringenin 7-*O*-glucoside), have also been reported in several *Salix* spp. bark extracts, including commercial ones [10,11,12,13]. These phenolic compounds have also demonstrated anti-hypertensive [14,15], cytoprotective [16], and antimicrobial [17] effects.

Considering the vast set of biological activities of *Salix* spp. bark extracts and their phenolic constituents, along with society’s increasing interest for natural components rather than synthetic ones, innovative food, nutraceutical, and cosmetic purposes can be envisaged. Actually, phenolic compounds have been increasingly used in the food industry as natural additives [18], as well as in the cosmetic field, including sunscreen and anti-aging cream formulations [19]. All of these applications are normally associated with their antioxidant activity, since phenolic compounds can disrupt the cascade oxidation reactions, either in food matrixes, allowing longer shelf life [18], or in dermatological preparations, preventing the oxidation of the other ingredients [19]. At the same time, the oral or topical administration of phenolic compounds can promote human wellbeing [18,19,20]. In this sense, alternative or complementary natural-based therapeutics have been researched for tackling current worldwide health problems, like hypertension [14,21] and multidrug-resistant bacterial infections [22,23].

Hypertension affects ca. 1.13 billion people, and is associated with premature mortality and disability [24]. Synthetic inhibitors of angiotensin-I converting enzyme (ACE) are the most used anti-hypertensive drugs; however, they can cause skin rashes, cough, angioedema, hypotension, renal disfunction, and other disturbing side effects [25]. Among phenolic compounds, flavan-3-ols, in particular procyanidins, have shown active ACE inhibition, being promising natural anti-hypertensive agents or coadjuvants [14].

Additionally, multidrug-resistant bacterial infections are a serious threat to public health, with an increased risk of morbidity and mortality, and financial burden on healthcare systems. Despite colonizing the skin of healthy humans, *Staphylococcus aureus* represents one of the leading causes of bacteremia, in addition to skin, soft tissue, and bone infections [26]. This Gram-positive bacterium can also lead to gastrointestinal illness, which comes from food contaminated by one of the 20 staphylococcal enterotoxins [27]. Phenolic compounds, like acetophenones and hydroxycinnamic acids, have exhibited anti-*S. aureus* effect [17,22,28].

Although *Salix* cultivation is more expanded in Northern Europe, several species of this genera are disseminated in Continental Portugal, namely *Salix atrocinerea* Brot., *S. fragilis* L., and *Salix viminalis* L., assuming a huge importance for the biodiversity and the soil stability in humid zones, within riparian ecosystems. Few works have evidenced the presence of phenolic constituents in the bark of these *Salix* species [29,30,31,32], but it is still missing a systematic approach, integrating the detailed phenolic composition, bioactivity, and biocompatibility of polar extracts of the individual species in question. Given the adequate edaphoclimatic conditions of Portugal for the selected *Salix* spp., this knowledge can boost their sustainable exploitation in Southern Europe, preserving the riparian ecosystem, enhancing the biodiversity, and contributing to rural development, in the context of the bioeconomy concept.

In the scope of our interest in bioprospecting *Salix* spp. bark [32], the present work aims to characterize the phenolic composition of *S. atrocinerea* Brot., *S. fragilis* L., and *S. viminalis* L. barks by ultra-high-performance liquid chromatography-diode array-tandem mass spectrometry (UHPLC-DAD-MS*^n^*), as well as to evaluate three key in vitro biological activities of their polar extracts, such as: (1) antioxidant activity, using two *in chimico* assays; (2) anti-hypertensive via ACE inhibitory effect; and (3) antibacterial effect against *S. aureus*. The cytotoxicity of the studied *Salix* spp. bark phenolic-containing extracts is also approached in three mammalian cell lines, namely, Caco-2, HaCaT, and L929 cell lines, towards potential safe food, nutraceutical, and cosmetic usages.

## 2. Materials and Methods

### 2.1. Chemicals

Dichloromethane (p.a., ≥99%), methanol (p.a., ≥99.8%), HPLC-grade methanol and acetonitrile were supplied by Fisher Scientific (Pittsburgh, PA, USA). Before UHPLC analysis, mobile phase solvents were previously filtered via a Solvent Filtration Apparatus 58061 from Supelco (Bellefonte, PA, USA). Acetic acid glacial (p.a., ≥99.5%) was purchased from Labkem (Madrid, Spain). Sodium carbonate (p.a., ≥99.9%) was obtained from Panreac AppliChem ITW Reagents (Barcelona, Spain). Gallic acid (≥97.5%), Folin–Ciocalteu’s phenol reagent (2 N), HPLC-grade water, formic acid (≥98%), catechin (>99%), eriodictyol (≥98%), naringenin (98%), procyanidin B1 (≥90%), procyanidin B2 (≥90%), quercetin (>98%), quercetin 3-*O*-galactoside (≥97%), 2,2-diphenyl-1-picrylhydrazyl free radical (DPPH•), 2,2′-azino-bis(3-ethyl-benzothiazoline-6-sulfonic acid) diammonium salt (ABTS), ascorbic acid (≥99.5%), angiotensin-I converting enzyme (ACE) (peptidyl-di-peptidase A, EC 3.4.15.1, 5.1 U mg^−1^), MEM non-essential amino acid solution, phenazine methosulfate, and 2,3-bis-(2-methoxy-4-nitro-5-sulfophenyl)-2*H*-tetrazolium-5-carboxanilide (XTT) were supplied by Sigma-Aldrich (Merck, Darmstadt, Germany). The intramolecularly quenched fluorescent tripeptide *o*-aminobenzoylglycyl-*p*-nitro-_L_-phenylalanyl-_L_-proline [Abz–Gly–Phe(NO_2_)–Pro] was purchased from Bachem Feinchemikalien (Bubendorf, Switzerland). Tris [tri(hydromethyl) aminomethane] was afforded by Fluka (Gmbh, Germany). Dulbecco’s Modified Eagle Medium (DMEM) high glucose and Penicillin-Streptomycin mixture were obtained from Lonza (Basel, Switzerland). Fetal bovine serum (FBS) was purchased from Biowest (Nuaillé, France). Piceol (≥98%), *m*-hydroxybenzoic acid (≥99%), tryptic soy broth and tryptic soy agar were afforded by Merck (Darmstadt, Germany). *p*-Hydroxybenzoic acid (>99%) was purchased from Fisher Scientific (Thermo Fisher Scientific Inc., Waltham, MA, USA). Naringenin 7-*O*-glucoside (≥99%) was supplied by Extrasynthese (Lyon, France).

### 2.2. Sampling of Salix spp. Barks

Branches from 8-year-old trees of *S. atrocinerea* Brot., *S. fragilis* L., and *S. viminalis* L. were collected nearby Aveiro (GPS coordinates 40°41′54.78” N, 8°36′3.23” W), from an industrial experimental plantation of The Navigator Company, in October 2017, and air-dried at room temperature until the biomass weight was stable [32]. Bark samples were hand-separated and ground using a hammer mill, in order to select the fraction with a granulometry lower than 1 mm.

### 2.3. Extraction of Phenolic Compounds

The lipophilic components were previously removed from the milled barks of the three *Salix* spp., as earlier reported [32]. Then, 2 g of lipophilic component free-dry bark was submitted to methanol/water/acetic acid (49.5:49.5:1) extraction, by stirring at 900 r.p.m. for 24 h, in the dark at room temperature, following a previously described approach [33]. After vacuum filtration through a glass filter of porosity 3 to separate the extract from the biomass, methanol was removed at 37 °C using a rotative evaporator, whilst water was removed by freeze-drying. *Salix* spp. extracts were prepared in triplicate, and the respective extractive yield (EY) was expressed as the percentage of dry bark. The extracts were then kept at room temperature and protected from the light, until the chemical analysis and the biological activity assays were performed.

### 2.4. Total Phenolic Content

The total phenolic content (TPC) of *Salix* spp. barks was determined using the Folin–Ciocalteu reagent, according to procedures carried out elsewhere [34,35], with some alterations. In a 96-well plate, 150 µL of Folin–Ciocalteu reagent previously diluted 1:10 (*v*/*v*) with water, and 120 µL of 75 g L^−1^ sodium carbonate aqueous solution were added to 30 µL of *Salix* spp. bark extracts, previously dissolved in methanol/water (1:1, *v*/*v*) and diluted with water, corresponding to 0.2 mg mL^−1^ of extract. After 60 min of incubation at room temperature and in the dark, the absorbance was recorded at 750 nm, against a blank containing 30 µL of water instead of the sample volume, in a Thermo Scientific Multiskan^TM^ FC microplate reader (Thermo Fisher Scientific Inc., Waltham, MA, USA). TPC was determined as gallic acid equivalents (GAE) using the linear regression equation (*y* = 0.0103*x* − 0.0276; *r*^2^ = 0.9995) obtained from the standard curve of gallic acid (5–100 µg mL^−1^), and expressed as grams of GAE per kilogram of dry bark and milligrams of GAE per gram of extract, according to Equations (1) and (2), as follows:

TPC (g GAE kg^−1^ of dry bark) = TPC (g GAE kg^−1^ of extract) × [EY (kg of extract kg^−1^ of dry bark)/100] (1)

TPC (mg GAE g^−1^ of extract) = [TPC (µg GAE mL^−1^) × dilution factor]/[extract concentration (g L^−1^) × 0.001] (2)

All of the assays were performed three times, each one in triplicate (*n* = 9).

### 2.5. Identification of Phenolic Compounds by UHPLC-DAD-MS^n^ Analysis

*Salix* spp. bark extracts were first dissolved in methanol/water (1:1, *v*/*v*), at 10 mg mL^−1^ and filtered using PTFE filters with 0.2 µm pore diameter. Extracts (10 µL) were injected in the UHPLC system equipped with an Accela 600 LC pump, an Accela autosampler (set at 16 °C), and an Accela 80 Hz photo diode array detector (DAD) (Thermo Fisher Scientific, San Jose, CA, USA). The separation of the extract components was developed in a Hypersil Gold RP C18 column (100 × 2.1 mm; 1.9 µm particle size) afforded by Thermo Fisher Scientific (San Jose, CA, USA), preceded by a C18 pre-column (2.1 mm i.d.) supplied by Thermo Fisher Scientific (San Jose, CA, USA), and both were kept at 45 °C. The binary mobile phase included (A) water/acetonitrile (99:1, *v*/*v*) and (B) acetonitrile, both containing 0.1% (*v*/*v*) formic acid. A gradient elution program was applied at a flow rate of 0.45 mL min^−1^, as follows: 1% B kept from 0 to 3 min; 1–31% B from 3 to 30 min; 31–100% B from 30 to 32 min, and 100–1% B from 32 to 36 min, keeping 1% B from 36 to 40 min for column re-equilibration. The chromatograms were recorded at 235, 280, and 370 nm and UV-Vis spectra from 210 to 600 nm.

The UHPLC system was coupled to a LCQ Fleet ion trap mass spectrometer (ThermoFinnigan, San Jose, CA, USA), equipped with an electrospray ionization (ESI) source. The ESI-MS was operated under the negative ionization mode with a spray voltage of 5 kV and capillary temperature of 320 °C. The flow rate of nitrogen sheath and auxiliary gas were 40 and 5 (arbitrary units), respectively. The capillary and tube lens voltages were set at −44 and −225 V, respectively. CID-MS*^n^* experiments were executed on mass-selected precursor ions in the range of *m*/*z* 100–2000. The isolation width of precursor ions was 1.0 mass units. The scan time was 100 ms and the collision energy was 35 arbitrary units, using helium as collision gas. The data acquisition was carried out by using Xcalibur^®^ data system (Thermo Finnigan, San Jose, CA, USA).

### 2.6. Quantification of Phenolic Compounds by UHPLC-UV Analysis

Standard curves were obtained through the UHPLC injection of catechin, *m*-hydroxybenzoic acid, naringenin, piceol, and quercetin standard solutions in HPLC grade methanol/water (1:1, *v*/*v*), with six concentrations ranging from 0.10 to 30.89 µg mL^−1^. The quantification of individual phenolic compounds was determined by using the linear regression equation (Table 1), obtained with the most similar standard compound. The limit of detection (LOD) and the limit of quantification (LOQ) were approached for each standard curve (Table 1), based on Equations (3) and (4), respectively, as follows:

LOD = (standard deviation of the ordinate intercept/slope of the linear regression) × 3(3)

LOQ = (standard deviation of the ordinate intercept/slope of the linear regression) × 10(4)

The quantitative analysis was performed in triplicate for each sample (*n* = 3).

### 2.7. Antioxidant Activity

#### 2.7.1. DPPH Free Radical Scavenging Effect

The DPPH• scavenging effect of *Salix* spp. bark extracts was measured according to a former method [34], with slight modifications for 96-well microplate scale. Ascorbic acid was used as the natural antioxidant reference. Briefly, stock solutions of extracts and ascorbic acid were firstly prepared in methanol/water (1:1, *v*/*v*). Then, 30 µL of 1 mM DPPH• methanolic solution was added to 75 µL of sample and 195 µL of methanol, in each microwell. The control was constituted by 270 µL of methanol and 30 µL of 1 mM DPPH• methanolic solution. The concentrations of extracts and ascorbic acid were tested in the 1–40 µg mL^−1^ and 0.5–20 µg mL^−1^ range, respectively. After a gentle mixing, the microplate was kept in the dark for 30 min, and the absorbance at 520 nm was thereafter read against the blank (methanol), using a Thermo Scientific Multiskan^TM^ FC microplate reader. The DPPH• scavenging effect percentage was calculated according to Equation (5):

DPPH• scavenging effect (%) = [(A_control_ − A_sample_)/A_control_] × 100 (5)

where A_control_ and A_sample_ are the absorbances at 520 nm of control and sample, respectively. The inhibitory concentration of extracts and ascorbic acid able to scavenge 50% of DPPH• (IC_50_) was calculated through the graph of scavenging effect percentage against concentration logarithm.

To compare the obtained results with the literature, the Antioxidant Activity Index (AAI) was determined according to Equation (6) [36]:

AAI = DPPH• final concentration (µg mL^−1^)/IC_50_ (µg mL^−1^) (6)

where DPPH• final concentration was 61.874 µg mL^−1^. All of the assays were performed three times, each one in triplicate (*n* = 9).

#### 2.7.2. ABTS Radical Cation Scavenging Effect

The ABTS radical cation (ABTS•^+^) scavenging effect of *Salix* spp. bark extracts was assayed based on the methodology reported elsewhere [34,37], which was adapted to the 96-well microplate scale. Ascorbic acid was used as the reference antioxidant. The ABTS•^+^ was first generated by mixing 7 mM ABTS and 2.45 mM potassium persulfate, and keeping the reactional mixture in the dark at room temperature for 16 h. Then, the ABTS•^+^ solution was diluted with methanol, in order to reach the absorbance value of 0.700 at 750 nm. Meanwhile, stock solutions of extracts and ascorbic acid were prepared in methanol/water (1:1, *v*/*v*). In each microwell, 250 µL of diluted ABTS•^+^ solution was added to 50 µL of sample, obtaining the 0.5–40 µg mL^−1^ and 0.5–16 µg mL^−1^ range for extracts and ascorbic acid, respectively. The control contained 250 µL of diluted ABTS•^+^ solution and 50 µL of methanol. Then, the microplate was kept in the dark for 30 min, and the absorbance at 750 nm was read against the blank (methanol) using the Thermo Scientific Multiskan^TM^ FC microplate reader. The ABTS•^+^ scavenging effect percentage was determined according to Equation (7):

ABTS•^+^ scavenging effect (%) = [(A_control_ − A_sample_)/A_control_] × 100 (7)

where A_control_ and A_sample_ are the absorbances at 750 nm of control and sample, respectively. The IC_50_ of extracts and ascorbic acid was determined from the scavenging effect percentage versus logarithm of concentration. All of the assays were performed three times, each one in triplicate (*n* = 9).

### 2.8. Angiotensin-I Converting Enzyme Inhibitory Activity

The ACE-inhibitory activity of *Salix* spp. bark extracts, at 625 µg mL^−1^, was measured by fluorescence using the method of Sentandreu and Toldrá [38], with some modifications [21]. The method consists in the ACE-catalyzed hydrolysis of a specific substrate [ABz–Gly–Phe(NO_2_)–Pro] to the fluorescent *o*-aminobenzoylglycine. Commercial ACE was diluted in 5 mL of 50% (*v*/*v*) glycerol aqueous solution, which was kept at −20 °C until use. Thereafter, the ACE solution was diluted (1:24) with 150 mM Tris buffer solution pH 8.3, containing 1 µM zinc chloride, for a final concentration of 42 mU mL^−1^. Then, 40 µL of ultrapure water or ACE working solution was added to each microplate well, and the volume was thereafter adjusted to 80 µL by adding ultrapure water to blank, control, or samples. A sample blank was also made. The enzymatic reaction was started by adding 160 µL of substrate solution (0.45 mM ABz–Gly–Phe(NO_2_)–Pro prepared in 150 mM Tris buffer pH 8.3, and containing 1.125 M sodium chloride), and then the mixture was incubated at 37 °C. The generated fluorescence was measured at 30 min using a Multidetection plate reader (Synergy H1, BioTek Instruments, Winooski, VT, USA). The assay was performed in a black 96-well microplate (Thermo Scientific Nunc, Roskilde, Denmark). Excitation and emission wavelengths were 350 and 420 nm, respectively. The inhibitory activity was calculated as the percentage decrease of ACE activity compared with the maximum ACE activity (control). All of the assays were performed two times, each one in duplicate (*n* = 4).

### 2.9. Inhibitory Effect Against Staphylococcus aureus Growth

The inhibitory effects of *Salix* spp. bark extracts were evaluated against the growth of a Gram-positive *S. aureus* strain (ATCC^®^ 6538). This bacterium was aseptically inoculated in tryptic soy broth, and grown at 37 °C under 120 r.p.m. for 24 h. Before the antibacterial test, the *S. aureus* density was adjusted to 0.5 McFarland in phosphate-buffered saline (PBS) solution, corresponding to 10^8^–10^9^ colony forming units (CFUs) mL^−1^. Then, the bacterial inoculum was incubated with the aqueous solutions of *Salix* spp. bark extracts at 37 °C for 24 h, obtaining the final concentrations of 625, 1250, and 2500 µg mL^−1^. The control containing only bacterial inoculum in PBS was also performed. Thereafter, the *S. aureus* bacterial density was determined by plating serial dilutions in tryptic soy agar. After 24 h of incubation at 37 °C, the antibacterial effect was assayed by determining the logarithm units of CFU mL^−1^ and comparing it with that of growth control group. In this study, the bacteriostatic and bactericidal effects were considered as the decrease of <3-log and ≥3-log in CFU mL^−1^, respectively, in comparison with the control inoculum [39]. All of the assays were performed three times, each one in duplicate (*n* = 6).

### 2.10. In Vitro Biocompatibility

#### 2.10.1. Mammalian Cell Lines

Three different cell lines were considered throughout this work, namely, Caucasian colon adenocarcinoma cells—Caco-2 (86010202, Sigma-Aldrich, St. Louis, MO, USA); human keratinocyte—HaCaT (300493, CLS, Eppelheim, Germany); and mouse fibroblast cells—L929 (NCTC) (ECACC 85103115). Caco-2 cells were maintained in DMEM high glucose supplemented with 10% (*v*/*v*) FBS, 1% (*v*/*v*) penicillin-streptomycin, and MEM non-essential amino acid solution. HaCaT and L929 cells were maintained using DMEM high glucose supplemented with 10% (*v*/*v*) FBS and 1% (*v*/*v*) penicillin-streptomycin. All of cell lines were incubated at 37 °C in a 5% (*v*/*v*) CO_2_ humidified atmosphere.

#### 2.10.2. Metabolic Inhibition via XTT Assay

Cells were detached using TrypLE Exress (Thermo Scientific, Waltham, MA, USA), seeded (1 × 10^4^ cells/well) into 96-well Nunclon Delta microplates (Thermo Scientific, Waltham, MA, USA), and incubated for 24 h. Afterwards, the culture media were carefully removed and replaced with *Salix* spp. bark extracts at 625, 1250, and 2500 µg mL^−1^ (sterile filtered). After incubation for 24 h, the cytotoxicity of the samples was evaluated using the XTT assay. Immediately before use, 10 μL of 10 mM phenazine methosulfate solution was added to 4 mL of 1 mg mL^−1^ XTT solution prepared in DMEM. Then, 25 μL of this mixture was added to each well, and the plates were, once again, incubated at 37 °C. After 2 h, the optical density at 485 nm was measured using a microplate reader (Synergy H1, Biotek Instruments, Winooski, VT, USA). Cells in culture medium were used as control, and wells without cells were used as blanks. The metabolic inhibition was determined according to the following Equation (8):

Metabolic inhibition (%) = [(A_control_ − A_sample_)/A_control_] × 100 (8)

where A_control_ and A_sample_ are the absorbances at 485 nm of control and sample, respectively. Five replicates for each condition were performed (*n* = 5).

### 2.11. Statistical Analysis

The statistical analysis was performed using the IBM^®^ SPSS^®^ Statistics Version 25 (IBM Corporation, New York, NY, USA). The EY, TPC, and the in vitro bioactivity assay data were analyzed through the one-way analysis of variance (ANOVA). Where differences existed, the source of the differences at *p* < 0.05 of significance level was identified by all pairwise multiple comparison procedures, through the Tukey’s honestly significant difference (HSD) post-hoc test. The Pearson’s correlation *r* values between TPC or phenolic compound abundances and the antioxidant activity IC_50_ values were also determined using the aforementioned software.

## 3. Results

### 3.1. Extractive Yield and Total Phenolic Content

In the present work, a methanol/water/acetic acid (49.5:49.5:1) solution was used for the extraction and chemical analysis of phenolic compounds in the studied *Salix* spp. barks, as it has proven to be suitable for the removal of these type of bioactive compounds from crops’ biomass [33].

The EY and TPC of *S. atrocinerea* Brot., *S. fragilis* L., and *S. viminalis* L. barks are summarized in Table 2.

*S. atrocinerea* Brot. bark showed the highest EY (15.1% of dry bark (*w*/*w*)), being significantly higher than EYs of *S. fragilis* L. and *S. viminalis* L. barks (*p* < 0.05). Considering the TPC determined using the Folin–Ciocalteu reagent, *S. atrocinerea* Brot. bark revealed the highest TPC, accounting for 44.47 g GAE kg^−1^ dry weight (dw). In terms of TPC expressed in mg g^−1^ of extract, *S. atrocinerea* Brot. bark extract also demonstrated the highest TPC (293.36 mg GAE g^−1^ of extract), but it was not statistically different from TPC of *S. viminalis* L. bark extract (*p* > 0.05). *S. fragilis* L. bark extract also presented considerable TPC, reaching 17.47 g kg^−1^ dw and 179.06 mg GAE g^−1^ of extract.

### 3.2. Phenolic Composition

#### 3.2.1. Identification of Phenolic Compounds

**Figure 1:**
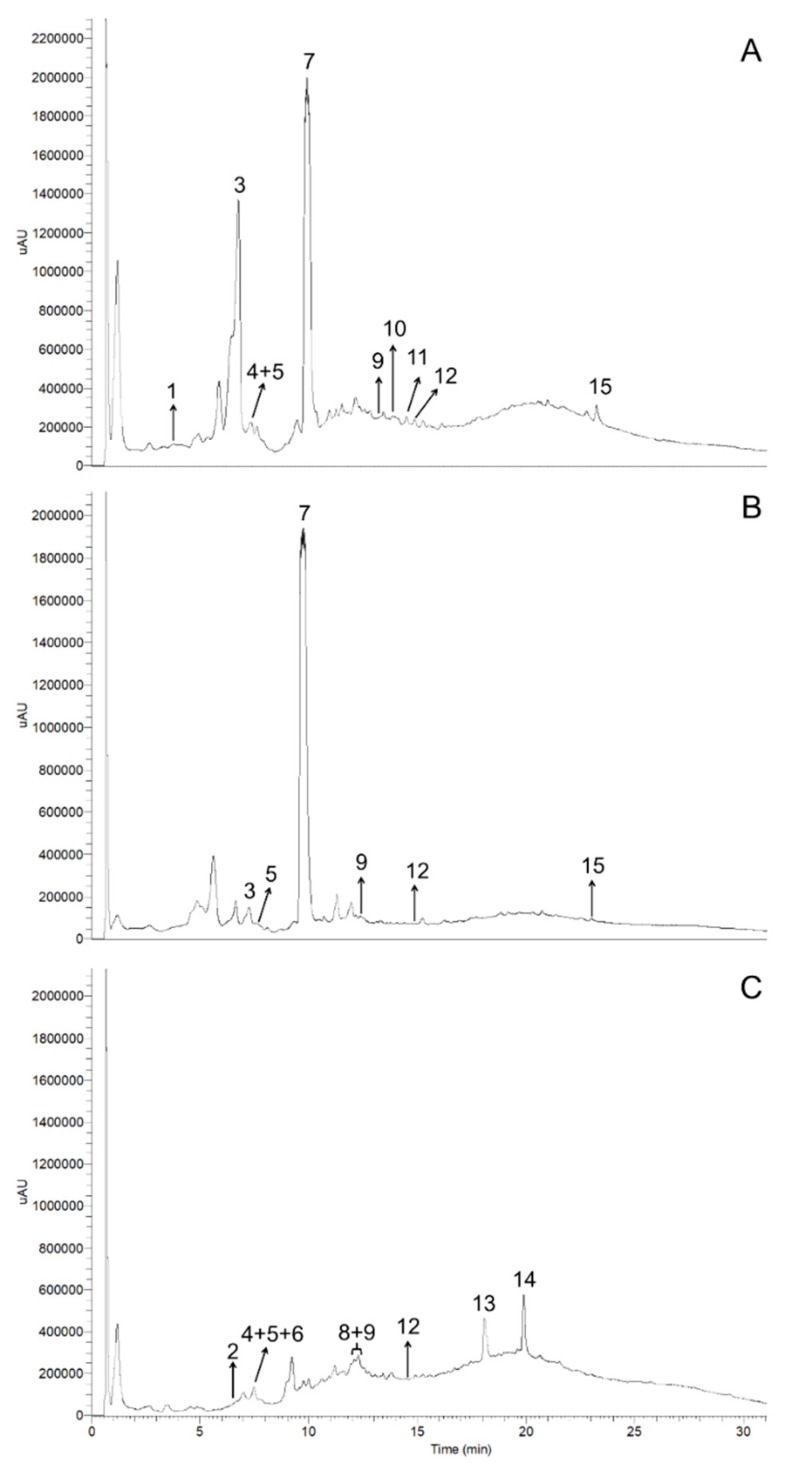
UHPLC-UV chromatograms of methanol/water/acetic acid (49.5:49.5:1) extracts, derived from (**A**) *Salix atrocinerea* Brot., (**B**) *Salix fragilis* L., and (**C**) *Salix viminalis* L. barks, recorded at 280 nm. The peak numbers correspond to those represented in Table 3 and Table 4 and Figure 2.

Figure 1 depicts the UHPLC-UV chromatograms of methanol/water/acetic acid extracts from *S. atrocinerea* Brot., *S. fragilis* L., and *S. viminalis* L. barks.

Fifteen phenolic compounds were detected in the studied *Salix* spp. bark polar extracts by HPLC-DAD-MS*^n^* analysis, as listed in Table 3 and explained thoroughly below.

##### Flavan-3-ols

**Figure 2:**
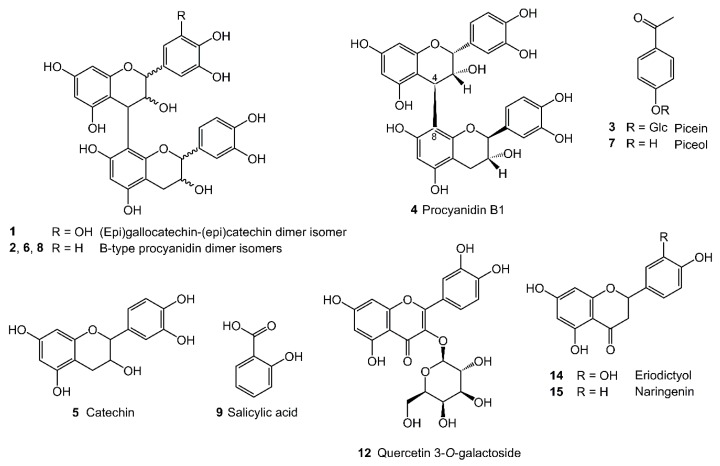
Proposed chemical structures for main phenolic compounds detected in the *Salix atrocinerea* Brot., *Salix fragilis* L., and *Salix viminalis* L. barks. Glc, glucosyl.

Compound 1 was tentatively assigned as a prodelphinidin dimer isomer, or (epi)gallocatechin-(epi)catechin dimer isomer (Figure 2), based on its UV spectrum (Figure S1A), and on the detection of the [M−H]^−^ ion at *m*/*z* 593 and MS*^n^* fragmentation (Table 3). The MS^2^ spectrum of the [M−H]^−^ ion showed the base peak at *m*/*z* 425, resulting from the retro-Diels-Alder fission of the C ring in the upper subunit ([M−H−168]^−^), as well as the product ion at *m*/*z* 407 given the sequent loss of a water molecule ([M−H−168−H_2_O]^−^) (see mass fragmentation 1 in Figure S2, in Supplementary Material) [40,41]. Moreover, the MS^2^ spectrum presented two product ions at *m*/*z* 303 and *m*/*z* 289 formed by the cleavage of the interflavanic linkage, corresponding to the quinone methide of the upper unit residue ([(epi)gallocatechin−3H]^−^) and the deprotonated ion of the lower unit residue ([(epi)catechin−H]^−^), respectively (see mass fragmentation 2 in Figure S2, in Supplementary Material). The product ion at *m*/*z* 289 can also be generated from the interflavanic fission of the product ion at *m*/*z* 467, after the C ring fission and the loss of a phloroglucinol moiety ([M−H−126]^−^) (see mass fragmentation 3 in Figure S2, in Supplementary Material) [43]. Additionally, the MS^3^ spectrum of the ion at *m*/*z* 289 showed the product ion at *m*/*z* 245, which is common to the mass fragmentation of catechin and epicatechin [42].

Compound 4 was identified as procyanidin B1 ((−)-epicatechin-(4β-8)-(+)-catechin) (Figure 2). The retention time, UV spectrum, the detection of the [M−H]^−^ ion at *m*/*z* 577, and the MS^2^ and MS^3^ fragmentations (Table 3) are in agreement with that of commercial standard, injected in the UHPLC-DAD-MS system, under the same experimental conditions.

Compounds 2, 6, and 8 were tentatively identified as B-type procyanidin dimer isomers (Figure 2) formed by two (epi)catechin units, due to their characteristic UV spectra (Figure S1B, in Supplementary Material), the detection of the [M−H]^−^ ion at *m*/*z* 577, and the MS*^n^* data (Table 3). The MS^2^ fragmentation of the [M−H]^−^ ion originated the product ion at *m*/*z* 425 (base peak) from the retro-Diels-Alder fission of the C ring ([M−H−152]^−^), which afforded the product ion at *m*/*z* 407 after a water molecule loss ([M−H−152−H_2_O]^−^) [43]. Furthermore, four characteristic product ions were detected, namely, at *m*/*z* 559 (loss of a water molecule), *m*/*z* 451 (heterocyclic C ring fission with the phloroglucinol moiety loss), as well as at *m*/*z* 289 and *m*/*z* 287, which resulted from the quinone methide fission of the interflavanic linkage between C and D rings [41,43]. Moreover, the MS^3^ spectrum of the ion at *m*/*z* 289 presented the characteristic product ions of catechin or epicatechin [42]. It was not possible to attribute the chemical structures of compounds 2, 6, and 8 to procyanidin B2, since none of their retention times were coincidental with the corresponding commercial standard. Since these compounds are procyanidin dimers of (epi)catechin units, there are six hypotheses of B-type procyanidins, namely, procyanidins B4, B5, and B8 [41], in addition to procyanidins B3, B6, and B7 detected earlier in *Salix* species [31], which can be suggested for their identification.

Compound 5 was identified as catechin (Figure 2 and Table 3) based on its UV spectrum, the detection of the [M−H]^−^ ion at *m*/*z* 289, and the characteristic MS^2^ data of the ion at *m*/*z* 289 [42], in addition to the injection of the commercial standard, at the same experimental conditions.

##### Acetophenones

Compound 3 presented a similar UV spectrum (Figure S1A, in Supplementary Material) to that of the picein (Figure 2), and afforded the ion at *m*/*z* 343, under the negative ionization [11], which corresponds to the formate adduct ion of that acetophenone ([M+HCOO]^−^) (Table 3). In addition to the [M−H]^−^ ion at *m*/*z* 297, two product ions were detected in the MS^2^ spectrum of the ion at *m*/*z* 343, namely, the base peak at *m*/*z* 135 resulting from the loss of a hexosyl unit of the [M−H]^−^ ion ([M−H−162]^−^), and the anion radical at *m*/*z* 120 which may be originated by homolytic fission of the methyl group from the aglycone ion ([M−H−162−CH_3_]^−^) [44]. In this sense, compound 3 was most likely assigned as picein, although the mass spectrometry analysis did not allow to discriminate the position of the *O*-glycosyl substituent. Nevertheless, the elution order of compound 3 relative to the commercial standard of piceol is in agreement with literature data [45].

Compound 7 was identified as piceol (Figure 2). The retention time, the UV spectrum, the detection of the [M−H]^−^ ion at *m*/*z* 135, and the MS^2^ fragmentation of this ion (Table 3), yielding the product ion at *m*/*z* 93 from the ketene loss ([M−H−42]^−^) [44], were concordant with that of commercial standard injected under the same experimental conditions. Although the product ion at *m*/*z* 120 would be expected in the mass fragmentation of the [M−H]^−^ ion at *m*/*z* 135 of compound 7, by comparison with the MS data of compound 3 and with literature [44], it was not found in the MS^2^ spectrum of the [M−H]^−^ ion obtained from the studied *Salix* extracts, or from the corresponding commercial standard. However, this fact does not hamper its unambiguous identification, since it has been corroborated with the retention time and MS data of the commercial standard.

##### Hydroxybenzoic Acids

Compound 9 was identified as *o*-hydroxybenzoic acid, commonly known as salicylic acid (Figure 2), presenting a UV spectrum (Figure S1B, in Supplementary Material) similar to that of salicylic acid [11], and the [M−H]^−^ ion at *m*/*z* 137 (Table 3). Additionally, the product ion at *m*/*z* 93 was found in the MS^2^ spectrum of the [M−H]^−^ ion, due to the CO_2_ loss ([M−H−44]^−^) [46], being concordant with the MS/MS data of salicylic acid, under the negative ionization mode [11]. Moreover, the retention time of compound 9 was different from those of commercial standards of *m*- and *p*-hydroxybenzoic acids injected in the HPLC-UV-MS system, under the same experimental conditions, thus being assigned as salicylic acid.

##### Flavanones

Compounds 10 and 11 were tentatively identified as two naringenin-*O*-hexoside isomers 1 and 2, respectively, whilst compound 15 was identified as naringenin (Figure 2) in the studied *Salix* spp. bark extracts (Table 3). Naringenin-*O*-hexoside isomers were assigned considering their UV spectra (Figure S1A, in Supplementary Material), the detection of the [M−H]^−^ ion at *m*/*z* 433, and the characteristic MS*^n^* fragmentation [11]. Indeed, the base peak of the MS^2^ spectrum of the aforementioned [M−H]^−^ ion was noted at *m*/*z* 271, which evidenced the loss of a hexosyl residue (−162 amu). Additionally, the MS^3^ spectrum of the ion at *m*/*z* 271 presented the characteristic product ions of naringenin [47]. Despite similar MS data, none of these compounds might be the chalcone isosalipurposide, since their absorption maxima wavelengths (274 and 277 nm) are completely different from that of the latter (370 nm) [11]. Furthermore, the retention times of compounds 10 and 11 were not concordant with that of commercial standard of naringenin 7-*O*-glucoside. Several hypotheses of naringenin-*O*-hexoside isomers can be proposed for their identification, including (+)- and (−)-naringenin 5-*O*-glucoside, as these have previously been found in *S. daphnoides* bark [11,12]. Nevertheless, the UV and MS data did not allow to distinguish the chemical structure of the glycosyl substituent and its position in the naringenin. Therefore, it was not possible to unequivocally identify compounds 10 and 11, only by UV spectra and MS data, being compounds’ isolation and chemical structure elucidation by NMR needed for their unambiguous identification. Compound 15 was identified as naringenin, based on the UV spectrum, the detection of the [M−H]^−^ ion at *m*/*z* 271, and the MS^2^ spectrum (Table 3). Furthermore, its identification was confirmed by running a commercial standard in the UHPLC-UV-MS system, at the same experimental conditions.

Compounds 13 and 14 were tentatively assigned as eriodictyol-*O*-hexoside isomer and eriodictyol (Figure 2), respectively, considering their UV spectra (Figure S1A, in Supplementary Material), the detection of the [M−H]^−^ ions at, respectively, *m*/*z* 449 and *m*/*z* 287, and the respective MS*^n^* fragmentation (Table 3) [33,48]. Regarding compound 13, the MS^2^ spectrum of the ion at *m*/*z* 449 showed the base peak at *m*/*z* 287, as a consequence of the hexosyl unit loss (−162 amu), whose MS^3^ spectrum demonstrated the characteristic product ions of the eriodictyol mass fragmentation [33]. Also, the earlier elution order of eriodictyol-*O*-hexoside in comparison to naringenin 7-*O*-glucoside is concordant with that reported in the literature [33]. Furthermore, the identification of eriodictyol was confirmed by comparing its retention time, molecular absorption UV spectrum, and MS data with that of a commercial standard.

##### Flavonols

Compound 12 was identified as quercetin 3-*O*-galactoside (Figure 2), taking into account its UV spectrum, the detection of the [M−H]^−^ ion at *m*/*z* 463, and the MS*^n^* data (Table 3) [49]. In fact, the MS^2^ spectrum of the [M−H]^−^ ion presented the base peak at *m*/*z* 301, whose product ion resulted from a hexosyl unit loss ([M−H−162]^−^. Furthermore, the MS^3^ spectrum of the ion at *m*/*z* 301 was concordant with that of quercetin [47]. The identification of compound 12 was unambiguously confirmed with the injection of the commercial standard of quercetin 3-*O*-galactoside in the HPLC-UV-MS system, under the same experimental conditions.

#### 3.2.2. Quantification of Identified Phenolic Compounds by UHPLC-UV Analysis

Table 4 depicts the contents of phenolic compounds present in the studied *Salix* spp. methanol/water/acetic acid extracts, expressed in mg kg^−1^ of dry weight (dw) and in mg g^−1^ of extract.

The total contents of identified phenolic compounds ranged from 490 mg kg^−1^ dw in *S. viminalis* L. bark (4.83 mg g^−1^ of extract) to 2871 mg kg^−1^ dw in *S. atrocinerea* Brot. bark (19.18 mg g^−1^ of extract).

Acetophenones represented the predominant phenolic compounds identified in *S. atrocinerea* Brot. bark extracts, accounting for 2155 mg kg^−1^ dw (14.42 mg g^−1^ of extract), as well as in *S. fragilis* L. bark extracts (1564 mg kg^−1^ dw and 16.15 mg g^−1^ of extract), mainly represented by piceol (**7**). Picein (**3**) was the second major phenolic compound identified in *S. atrocinerea* Brot. bark extracts, while it was present in *S. fragilis* L. bark extracts at a much lower content (up to 29-fold).

*S. atrocinerea* Brot. bark also contained the highest flavan-3-ol content, representing 617 mg kg^−1^ dw (4.10 mg g^−1^ of extract), and being up to 4-fold higher than in *S. fragilis* L. bark. In particular, procyanidin B1 (**4**) and catechin (**5**) were the major flavan-3-ols present in *S. atrocinerea* Brot. bark extracts. Salicylic acid (**9**) was majorly present in *S. viminalis* L. bark extracts (200 mg kg^−1^ dw and 2.00 mg g^−1^ of extract). Additionally, *S. viminalis* L. bark extracts demonstrated the highest flavanone content (103 mg kg^−1^ dw and 1.00 mg g^−1^ of extract), being 21-fold higher relative to *S. fragilis* L., whereas *S. atrocinerea* Brot. bark extracts showed the highest flavonol content (up to 6-fold higher when compared with *S. fragilis* L. bark extracts).

Taking in account the differentiated phenolic composition of *Salix* spp. bark extracts, in addition to the reported antioxidant, anti-hypertensive, and antimicrobial effects of the identified phenolic compounds [9,14,15,17], three in vitro biological activities were evaluated, as follows: (1) antioxidant activity, via scavenging effects against DPPH• and ABTS•^+^ free radicals; (2) anti-hypertensive potential, via the inhibitory effect on ACE enzymatic activity; and (3) antibacterial action via inhibitory effect against *S. aureus*. Finally, to ensure that the extracts can be safely used, for instance in food, nutraceutical, or cosmetic formulations, the in vitro biocompatibility of *Salix* spp. bark extracts was also conducted in three mammalian cell lines, namely, Caco-2, HaCaT, and L929 cell lines.

### 3.3. In Vitro Bioactivity of Salix spp. Bark Polar Extracts

#### 3.3.1. Antioxidant Activity

The antioxidant activity of *Salix* spp. bark polar extracts was assessed through the DPPH• and ABTS•^+^ scavenging effect assays, as denoted in Table 5.

*S. atrocinerea* Brot. bark extracts were the most active in scavenging the DPPH• and ABTS•^+^ (IC_50_ of 10.98 and 5.58 µg mL^−1^, respectively), although their IC_50_ were not statistically different from *S. viminalis* L. extracts (*p* > 0.05), when using Tukey’s HSD test for pairwise multiple comparison procedure. On the other hand, *S. fragilis* L. bark extracts presented the lowest antioxidant effect, with IC_50_ of 23.62 and 10.24 µg mL^−1^, respectively in the DPPH• and ABTS•^+^ scavenging activities. Particularly in what concerns to the ABTS•^+^ scavenging effect, the IC_50_ of *S. fragilis* L. extracts was not statistically different from that of *S. viminalis* L. extracts (*p* > 0.05), using Tukey’s HSD test. Comparing the DPPH• and ABTS•^+^ scavenging effects of *Salix* spp. bark extracts with that of ascorbic acid, *S. atrocinerea* Brot. was 2.8- and 1.7-fold less effective than the natural antioxidant standard in the respective assays, but with no statistical differences using the Tukey’s HSD test (*p* > 0.05) were observed. Furthermore, the antioxidant activity of *S. fragilis* L. and *S. viminalis* L. bark extracts was significantly weaker than that of ascorbic acid (*p* < 0.05) in both assays. According to the AAI rank suggested by Scherer and Godoy [36], all *Salix* spp. bark extracts presented very strong antioxidant activity in the DPPH• assay.

#### 3.3.2. Angiotensin-I Converting Enzyme Inhibitory Activity

**Figure 3:**
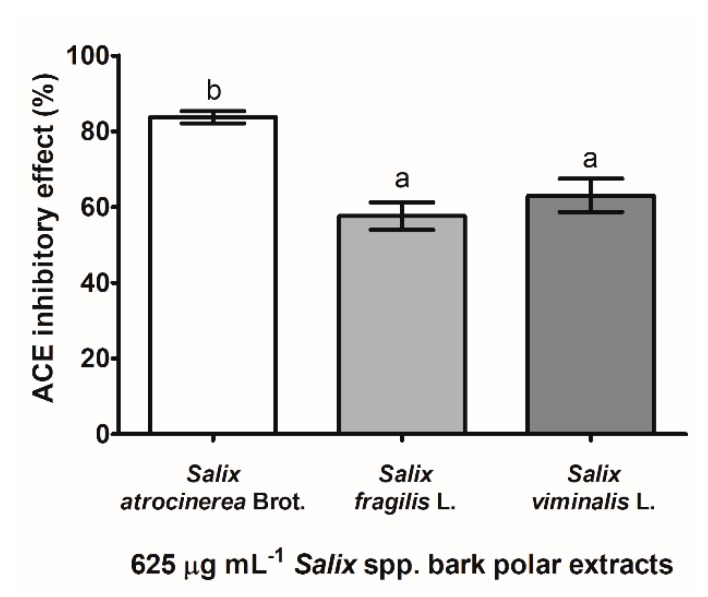
Inhibitory effect of 625 µg mL^−1^ *Salix atrocinerea* Brot., *Salix fragilis* L., and *Salix viminalis* L. bark extracts against the angiotensin I-converting enzyme (ACE). Each column and bar represents the mean and the standard deviation, respectively (*n* = 4). Columns with different minor case letters (a, b) are statistically different (one-way ANOVA, followed by Tukey’s HSD test, *p* < 0.05).

The inhibitory effects of *Salix* spp. bark extracts were assessed at 625 µg mL^−1^ against the enzymatic activity of ACE, as illustrated in Figure 3.

Hence, *S. atrocinerea* Brot. bark polar extracts largely decreased the enzymatic activity of ACE (84 ± 2% of inhibition), being 1.5- and 1.3-fold more active than *S. fragilis* L. (58 ± 4%) and *S. viminalis* L. (63 ± 4%) bark extracts, respectively (*p* < 0.05). To the best of our knowledge, the inhibitory effect of *Salix* spp. bark extracts on ACE was evaluated herein for the first time, showing the promising potential for the anti-hypertensive purpose.

#### 3.3.3. Inhibitory Effect against *S. aureus* Growth

**Figure 4:**
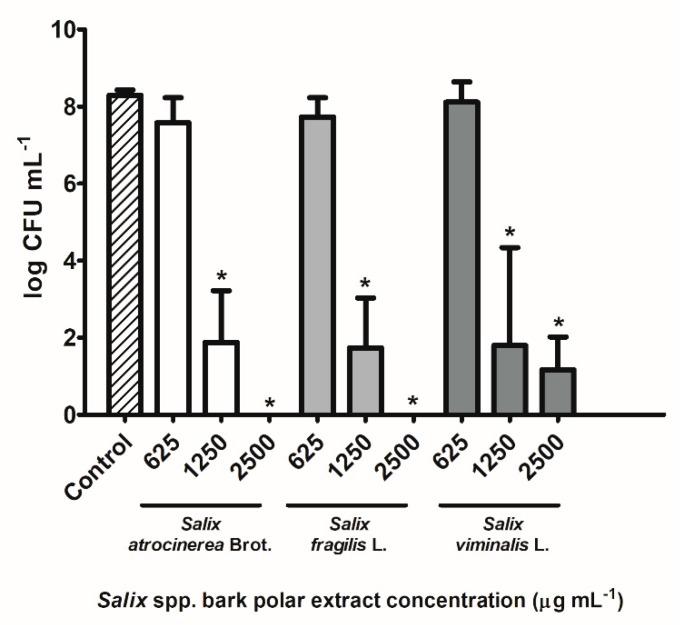
Bacterial density expressed as log CFU mL^−1^ of *Staphylococcus aureus* ATCC^®^ 6538, after 24 h of incubation with 625, 1250, and 2500 µg mL^−1^ of *Salix atrocinerea* Brot., *Salix fragilis* L., and *Salix viminalis* L. bark polar extracts. Growth bacterial control is also depicted. Each column and bar represents the mean and the standard deviation, respectively (*n* = 6). Columns with the symbol * are statistically different from the growth control (one-way ANOVA, followed by Tukey’s HSD test, *p* < 0.05). CFU, colony forming unit.

The inhibitory effects of *S. atrocinerea* Brot., *S. fragilis* L., and *S. viminalis* L. bark extracts were tested for 24 h against the growth of the Gram-positive bacterium *S. aureus*, as depicted in Figure 4.

All the 24 h-treatments reduced the bacterial growth in a concentration-dependent manner, but statistical differences were not found with 625 µg mL^−1^ *Salix* spp. bark extracts, when compared with the growth control group (*p* > 0.05). Notwithstanding, all *Salix* spp. bark extracts tested at 1250 and 2500 µg mL^−1^ reduced significantly the bacterial growth regarding to the control group (*p* < 0.05), exhibiting bactericidal effects, as caused ≥3 log CFU mL^−1^ decrease. More specifically, 1250 µg mL^−1^ *S. atrocinerea* Brot. led to 6 log CFU mL^−1^ decrease, whereas *S. fragilis* L. and *S. viminalis* L. extracts to 7 log CFU mL^−1^ reduction. It is noteworthy to highlight that no bacterial colonies were detected after the treatments with 2500 µg mL^−1^ *S. atrocinerea* Brot. and *S. fragilis* L. extracts (8 log CFU mL^−1^ reduction), whilst *S. viminalis* L. extracts decreased significantly 7 log CFU mL^−1^, at the same concentration, in comparison with the control (*p* < 0.05).

### 3.4. In Vitro Biocompatibility of Salix spp. Bark Polar Extracts

**Figure 5:**
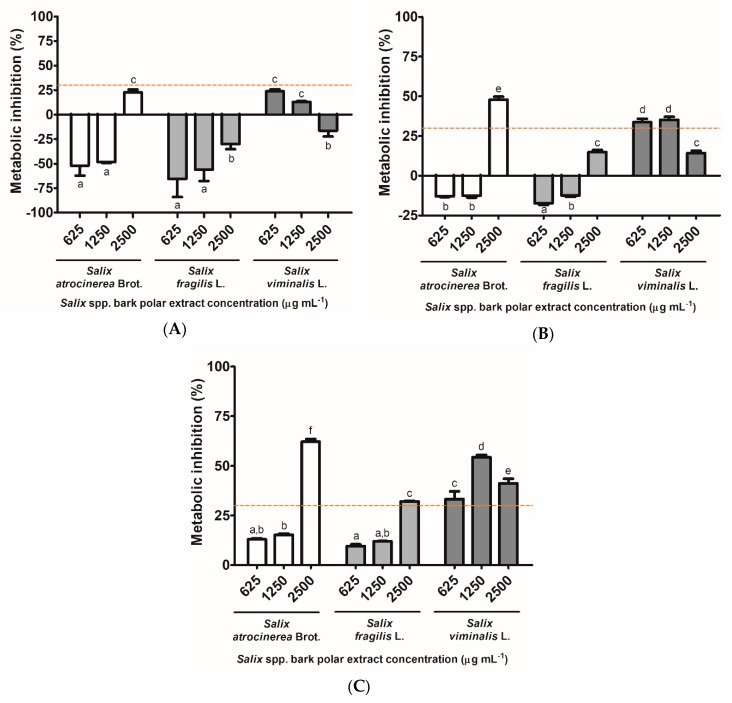
Metabolic inhibition of *Salix atrocinerea* Brot., *Salix fragilis* L., and *Salix viminalis* L. bark polar extracts at 625, 1250, and 2500 µg mL^−1^ for 24 h against three mammalian cell lines, namely: (**A**) human colorectal adenocarcinoma Caco-2 cells; (**B**) human keratinocyte HaCaT cells; and (**C**) mouse fibroblast L929 cells. Each column and bar represents the mean and the standard deviation, respectively (*n* = 5). Columns with different minor case letters (a–e) are statistically different (one-way ANOVA, followed by Tukey’s HSD test, *p* < 0.05).

The cytotoxicity of *S. atrocinerea* Brot., *S. fragilis* L., and *S. viminalis* L. bark extracts was assayed at the 625–2500 µg mL^−1^ range for 24 h in Caco-2, HaCaT, and L929 cells (Figure 5).

According to the international standard ISO 10993-5 for the biological evaluation of medical devices (part 5: Tests for in vitro cytotoxicity), the threshold value for a sample to be cytotoxic is a metabolic inhibition of 30%. As such, as can be seen in Figure 5A, none of the tested *Salix* spp. bark extracts exerted a cytotoxic effect against Caco-2 cells. In fact, some of the concentrations appeared to stimulate the mitochondrial metabolism of this cell line. For HaCaT cells (Figure 5B), none of the tested concentrations of *S. fragilis* L. bark extracts exhibited a cytotoxic effect against this cell line, with the *S. atrocinerea* Brot bark extracts at 625 and 1250 μg mL^−1^ demonstrating the same effect. In fact, only the highest concentration of *S. atrocinerea* Brot. exhibited a clear cytotoxic effect, as well as the two lower concentrations of *S. viminalis* L. (625 and 1250 μg mL^−1^) resulted in a metabolic inhibition which is close to the threshold value, therefore requiring further studies (namely, to study the production of apopototic markers), particularly as *S. viminalis* L. bark extract at 2500 µg mL^−1^ did not exert a cytotoxic effect. The mouse fibroblast L929 cells (Figure 5C) appeared to be more susceptible to the presence of the extracts than the remaining tested cell lines, with the highest concentration of *S. atrocinerea* Brot. and *S. fragilis* L. extracts, along with all concentrations of *S. viminalis* L. extracts, resulting in metabolic inhibitions above 30%. Overall, it is important to mark that, at 625 and 1250 μg mL^−1^, *S. atrocinerea* Brot. and *S. fragilis* L. extracts did not exert a cytotoxic effect against any of the tested cell lines.

## 4. Discussion

The present work describes, for the first time, the detailed phenolic characterization, as well as the in vitro bioactivity and biocompatibility, of *S. atrocinerea* Brot., *S. fragilis* L., and *S. viminalis* L. bark polar extracts, aiming at their sustainable and safer bioprospection towards novel and innovative food, nutraceutical, and/or cosmetic applications.

Using methanol/water/acetic acid (49.5:49.5:1) solution for the phenolic compounds’ extraction from the studied *Salix* spp. barks, the EY ranged from 9.7% in *S. fragilis* L. to 15.1% (*w*/*w*) in *S. atrocinerea* Brot. barks (Table 2). Comparing with the literature data for the distinct *Salix* species and extraction solvents, the EY of *S. atrocinerea* Brot. bark was 1.2-fold higher than that of 70% (*v*/*v*) acetone extract of *S. psammophila* bark, but 1.8- and 2.0-fold lower than the same kind of extracts of *S. sachalinensis* and *S. pet-susu* bark, respectively [50].

Moreover, *S. atrocinerea* Brot. bark showed the highest TPC (Table 2), accounting for 44.47 g GAE kg^−1^ dw and 293.36 mg GAE g^−1^ of extract, but with no statistical significance (*p* > 0.05) when comparing its TPC expressed in mg GAE g^−1^ of extract with that of *S. viminalis* L. bark. *S. fragilis* L. bark also demonstrated considerable TPC, i.e., 17.47 g GAE kg^−1^ dw and 179.06 mg GAE g^−1^ of extract. TPC of *S. atrocinerea* Brot. bark was 2.8-fold higher relative to *S. psammophila* bark [50], but lower than *S. subserrata* Willd. (up to 1.8-fold) [51], *S. aegyptiaca* L. (up to 4.8-fold) [52], *S. sachalinensis* (2.3-fold), and *S. pet-susu* (2.5-fold) barks [50]. Nevertheless, some caution should be taken in these comparisons, since *Salix* barks from different species and geographical origins were used, in addition to the different extraction media and methodologies applied, obviously affecting EY and TPC.

Fifteen phenolic compounds were found in bark polar extracts of the three *Salix* spp. in study, by UHPLC-UV-MS*^n^* (Figure 1 and Table 3), namely six flavan-3-ols (**1**, **2**, **4**–**6**, and **8**), two acetophenones (**3** and **7**), a hydroxybenzoic acid (**9**), five flavanones (**10**, **11**, **13**–**15**), and a flavonol (**12**) (Figure 2). Regarding to flavan-3-ols, procyanidin B1 (**4**) and catechin (**5**) have been previously detected in *S. viminalis* L. bark [31,53]. In addition to procyanidin B1 (**4**), three other B-type procyanidin dimer isomers (**2**, **6**, and **8**) were also detected in *S. viminalis* L. bark. However, no B-type procyanidin dimer isomers were herein identified in *S. fragilis* L. bark, contrarily to what reported by Pobłocka-Olech and Krauze-Baranowska [31], which may be related not only with the extraction methodology and analytical techniques, but also with the geographical origin, climatic conditions, season, plant age, and genotype-phenotype associations. Still, it is remarkable the number of B-type procyanidins besides procyanidin B1 that have already been identified in several *Salix* spp., namely, procyanidins B3, B6, and B7 [31], which may potentiate interesting applications of this biomass in the food and health fields, due to their vast biological effects, including antioxidant.

This work also evidences, for the first time, the identification of catechin (**5**) in *S. atrocinerea* Brot. and *S. fragilis* L. barks, as well as a prodelphinidin dimer isomer (**1**) and procyanidin B1 (**4**) in *S. atrocinerea* Brot. bark. Considering the acetophenones, picein (**3**) was identified here, for the first time, in *S. atrocinerea* Brot. and *S. fragilis* L. barks. Furthermore, this phenolic compound has been described in the bark of other *Salix* species, namely, *S. daphnoides* [11], *S. purpurea* [13], and willow hybrid “Karin” [10]. In the same sense, piceol (**7**) and salicylic acid (**9**) were also found in the studied extracts of the two aforementioned *Salix* species, being recently identified in the respective lipophilic fractions [32].

In what concerns flavanones, two naringenin-*O*-hexoside isomers (**10** and **11**) were herein identified for the first time as phenolic constituents of the three analyzed *Salix* spp. barks. Naringenin (**15**) has recently been detected in *S. fragilis* L. bark [54], but it is revealed, for the first time in this work, as a phenolic component of *S. atrocinerea* Brot. bark. It is worth underlining that (+)- and (−)-naringenin 5-*O*-glucoside, naringenin 7-*O*-glucoside, and naringenin (**15**) have also been found in the barks of *S. daphnoides* and *S. purpurea* [11,12,13,30]. Eriodictyol-7-*O*-glucoside and eriodictyol (**14**) have been isolated from a commercial willow bark extract, with *S. fragilis* L. bark included in the formulation [55,56]. However, to the best of our knowledge, an eriodictyol-*O*-hexoside isomer (**13**) and eriodictyol (**14**) were reported herein for the first time in *S. atrocinerea* Brot. and *S. viminalis* L. barks. Regarding flavonols, only quercetin 3-*O*-galactoside (**12**) was found in *S. atrocinerea* Brot., *S. fragilis* L., and *S. viminalis* L. barks, being described in these raw materials for the first time.

Considering their quantitative analysis (Table 4), the total contents of identified phenolic compounds varied between 490 mg kg^−1^ dw in *S. viminalis* L. bark and 2871 mg kg^−1^ dw in *S. atrocinerea* Brot. bark. Comparing these results with TPC (Table 2), not only they did not follow the same trend as TPC, but they also corresponded to a minor part of TPC (ca. 2–10%), similar to what has been observed with other shrubs [57]. This may be explained by an array of extracts’ components other than phenolic compounds that can react with the Folin–Ciocalteu’s reagent in alkaline medium, including sugars and organic acids, among others [58].

Acetophenones were the main phenolic constituents of *S. atrocinerea* Brot. and *S. fragilis* L. barks, accounting for 2155 and 1564 mg kg^−1^ dw, respectively. Piceol (**7**) abundance is clearly higher in both *Salix* bark polar extracts than in the respective lipophilic fractions [32], while picein (**3**) content is up to 10.5-fold higher than in *S. caprea* L. bark [59], but up to 5.7-fold lower than that described for *S. phylicifolia* L., *S. myrsinifolia* Salisb., and *S. pentandra* L. barks [60], which may be related to the aforementioned factors.

Flavan-3-ols were also present at considerable contents in *S. atrocinerea* Brot. bark, accounting for 617 mg kg^−1^ dw, followed by *S. viminalis* L. and *S. fragilis* L. barks, but being 4.5-fold lower than the one reported for *S. viminalis* L. bark [53]. Minor abundances of flavanones and flavonols were detected in the studied *Salix* species.

Due to the antioxidant [9,61], anti-hypertensive [14,15], and antimicrobial [17] effects exhibited by the analyzed phenolic compounds, *Salix* spp. bark polar extracts were evaluated for these biological activities.

The antioxidant activity of *Salix* spp. bark polar extracts was assessed through the scavenging activity against DPPH• and ABTS•^+^ radicals (Table 5). Ascorbic acid was used as a natural antioxidant reference. Indeed, *S. atrocinerea* Brot. extracts were more active in scavenging DPPH• and ABTS•^+^ radicals, although with no statistical difference (*p* > 0.05) when using Tukey’s HSD test, in comparison with *S. viminalis* L. extracts. Despite evidencing higher IC_50_ values, the antioxidant activity of *S. atrocinerea* Brot. extracts was not significantly different from ascorbic acid in both assays (*p* > 0.05), using Tukey’s HSD test. Nevertheless, the DPPH• scavenging effect of *Salix* spp. bark extracts can be considered as very strong, according to the AAI (Table 5) [36]. Yet, taking the AAI in consideration, *S. atrocinerea* Brot. bark extracts are 8.5- and 2.3-fold stronger than, respectively, *S. alba* L. bark 70% methanol [62] and *S. aegyptiaca* L. bark ethanol extracts [52], but slightly weaker (1.4-fold) than *S. subserrata* Willd. bark 80% methanol extracts [51]. Comparing the ABTS•^+^ scavenging effect of the studied *Salix* spp. bark extracts with other species, all are considerably much stronger than the water extracts of *S. myrsinifolia* and *S. purpurea* barks (IC_50_ values of 7 and 20 mg mL^−1^, respectively) [63]. Bridging the phenolic composition with the antioxidant activity of the studied *Salix* spp. bark extracts, the strongest significant correlation in each assay was found between flavan-3-ol content and DPPH• scavenging effect (Pearson’s correlation, *r* = −0.637; *p* < 0.033), and between flavan-3-ol abundance and ABTS•^+^ scavenging effect (Pearson’s correlation, *r* = −0.669; *p* < 0.024). Actually, flavan-3-ols like procyanidins have demonstrated strong DPPH• and ABTS•^+^ scavenging effects [64,65]. A significant correlation was also achieved between the flavonol abundance and ABTS•^+^ scavenging effect (Pearson’s correlation, *r* = −0.647; *p* < 0.030). Moreover, flavanone and flavonol contents could be slightly correlated with DPPH• scavenging effect (Pearson’s correlation, *r* values of −0.580 and −0.543 respectively), but they were not statistically significant (*p* > 0.05). TPC was also significantly correlated with the DPPH• scavenging effect (Pearson’s correlation, *r* = −0.665; *p* < 0.025). Although there was a smooth correlation between TPC and ABTS•^+^ scavenging effect (Pearson’s correlation, *r* = −0.546), it was not significant (*p* > 0.05). The hypothesis of synergisms occurring between flavan-3-ols and other phenolic compounds, or even other extracts’ components, should indeed be placed.

*Salix* spp. bark phenolic-containing extracts were investigated for their anti-hypertensive potential (Figure 3), through the inhibitory effects against ACE. Hence, all *Salix* spp. bark extracts at 625 µg mL^−1^ diminished the enzymatic activity of ACE, ranging from 58 to 84% of inhibition, with *S. atrocinerea* Brot. bark extracts as the most effective (*p* < 0.05). To the best of our knowledge, the inhibitory effect of *Salix* spp. bark extracts on ACE was evaluated herein for the first time. In fact, the ACE inhibitory effect has been poorly approached for polar extracts of woody plants, as assayed with 70% ethanol extracts of *Populus tremula* L. (Salicaceae) bark, *Betula pendula* Rot. (Betulaceae) buds, and *Quercus robur* L. (Fagaceae) bark, at 100 µg mL^−1^, and ranging from 11 to 28% of inhibition, respectively [66]. Flavan-3-ols may be strongly involved in the inhibitory effect of *S. atrocinerea* Brot. bark extracts, since these phenolic compounds have shown an interesting ACE inhibitory activity [14,15].

The inhibitory effect of *Salix* spp. bark polar extracts was also evaluated against the growth of the bacterium *S. aureus* ATCC^®^ 6538 (Figure 4). Thus, the 24 h-treatments with *S. atrocinerea* Brot., *S. fragilis* L., and *S. viminalis* L. bark polar extracts reduced *S. aureus* growth, in a concentration-dependent manner (Figure 4), but statistical differences were not found at 625 µg mL^−1^ *Salix* spp. bark extracts, when compared with the control group (*p* > 0.05). On the other hand, *Salix* spp. bark extracts at 1250 and 2500 µg mL^−1^ significantly decreased the *S. aureus* growth compared to the control group (*p* < 0.05), with a 6–8 log CFU mL^−1^ range reduction, meaning that all extracts were bactericidal for this microorganism. Previous studies have demonstrated the anti-*S. aureus* potential of other *Salix* spp. bark extracts, including *S. mucronate* L. bark ethyl acetate (minimum inhibitory concentration (MIC) of 3125 µg mL^−1^) [67] and *S. capense* extracts (5–1000 µg mL^−1^ MIC range) [68]. Phenolic compounds, like picein (**3**) and piceol (**7**), have also exhibited inhibitory effect against *S. aureus* growth, with MICs of 650 and 900 µg mL^−1^, respectively [17]. Synergisms between phenolic and other extracts’ constituents may have occurred, but a bioactive-guided fractionation should be conducted.

For future safe usage of *Salix* spp. bark phenolic-containing extracts, especially in what regards to food, nutraceutical, or cosmetic applications, their in vitro biocompatibility was addressed in human colorectal adenocarcinoma Caco-2 cells, human keratinocyte HaCaT cells, and mouse fibroblast L929 cells, analyzing the 24 h-inhibitory effects on cell metabolism (Figure 5). Globally, *S. atrocinerea* Brot. and *S. fragilis* L. bark extracts did not present cytotoxic effects at 625 and 1250 µg mL^−1^ against the three cell lines, as the metabolic inhibition was lower than 30%. Regarding to *S. viminalis* L. bark extracts, they were not cytotoxic against Caco-2 cells at all tested doses (Figure 5A), and against HaCaT cells at the highest concentration (Figure 5B), although all the tested concentrations suppressed the metabolism of L929 cells (Figure 5C) more than 30%. The cytotoxic potential of these *Salix* spp. bark extracts may be associated with some of the identified phenolic compounds, such as naringenin (**15**) and catechin (**5**), which have previously shown mild cytotoxic effect against H1299 human lung cancer cells after 24 h-incubation [69]. In this sense, the proposed applications of *Salix* spp. bark polar extracts should be tuned based on their non-cytotoxic concentrations.

In summary, *Salix* spp. bark polar extracts evidenced strong antioxidant activity, promising anti-hypertensive potential and effective antibacterial action against *S. aureus*. Notwithstanding, some attention should be paid to the non-cytotoxic concentrations of these extracts, being necessary to plan applications of *S. atrocinerea* Brot. and *S. fragilis* L. extracts for non-cytotoxic doses, and to better understand the cytotoxic effect of *S. viminalis* L. extracts. Moreover, an activity-guided fractionation is further needed in order to clarify the main bioactive constituents of *Salix* spp. bark extracts. Alternative extraction methodologies and solvents, like ultrasound and microwave-assisted extractions, and deep eutectic solvents should be considered for the extraction of *Salix* spp. bark phenolic compounds, intending their promising applicability in food, nutraceutical, and dermatological fields, towards the sustainable exploitation of this biomass and, at the same time, contributing for the biodiversity and rural profits.

## 5. Conclusions

The present study evidences, for the first time, the detailed phenolic characterization of three Portuguese *Salix* spp. bark samples, namely, *S. atrocinerea* Brot., *S. fragilis* L., and *S. viminalis* L., as well as the in vitro health-promoting potential of these polar extracts, such as antioxidant, anti-hypertensive, and antibacterial effects, and biocompatibility. Fifteen phenolic compounds were revealed in *Salix* spp. barks, by UHPLC-UV-MS*^n^*, being two flavan-3-ols, an acetophenone, five flavanones, and a flavonol, detected for the first time in the studied *Salix* spp. barks. *S. atrocinerea* Brot. extracts demonstrated the highest total content of identified phenolic compounds (2871 mg kg^−1^ dw and 19.18 mg g^−1^ of extract), including acetophenones (2155 mg kg^−1^ dw and 14.42 mg g^−1^ of extract) and flavan-3-ols (617 mg kg^−1^ dw and 4.10 mg g^−1^ of extract). In what concerns the *in vitro* biological activity, *Salix* spp. bark extracts exhibit strong DPPH• and ABTS•^+^ free radical scavenging effects (5.58–23.62 µg mL^−1^ IC_50_ range) and ACE inhibitory effects (58–84% of inhibition). Moreover, all extracts at 1250–2500 µg mL^−1^ exhibited bactericidal activity (6–8 log CFU mL^−1^ reduction) against *S. aureus*. The three in vitro biological activities may be mainly related to the presence of flavan-3-ols and acetophenones, but synergism effects may occur between these compounds and other extracts’ phenolic subclasses or constituents. Indeed, a bioactivity-guided fractionation should be further performed to clearly elucidate the bioactive component(s). Nonetheless, some caution should be taken in the safe use of these extracts, considering their non-cytotoxic doses. Overall, these promising insights can foster the economic valorization of the three studied Portuguese *Salix* spp., as raw materials of phenolic-containing extracts with an array of biological activities, towards innovative and novel food, nutraceutical, or cosmetic applications, along with the energy generation, being in line with the biorefinery concept.

## Figures and Tables

**Table 1:** Standard data used for the HPLC-UV quantification of phenolic compounds present in methanol/water/acetic acid (49.5:49.5:1) extracts of *Salix* spp. bark.

Standard Compound	λ (nm) ^A^	Concentration Range (µg mL^−1^)	Linear Regression Equation ^B^	*r* ^2^	LOD (µg mL^−1^)	LOQ (µg mL^−1^)
Catechin	280	0.10–30.29	*y* = 93621*x* + 17212	0.9998	0.52	1.74
*m*-Hydroxybenzoic acid	235	0.51–30.89	*y* = 245747*x* + 909936	0.9929	3.42	11.40
Naringenin	280	0.11–21.17	*y* = 398130*x* + 61541	0.9990	0.87	2.89
Piceol	280	0.30–18.23	*y* = 765733*x* + 59082	0.9992	0.68	2.25
Quercetin	370	0.10–19.21	*y* = 320421*x* − 99949	0.9989	0.85	2.83

^A^ Wavelength used in the quantitative analysis; ^B^ *y* = peak area, *x* = concentration in µg mL^−1^. LOD, limit of detection; LOQ, limit of quantification.

**Table 2:** Extractive yield (EY) and total phenolic content (TPC) of methanol/water/acetic acid (49.5:49.5:1) extracts of *Salix atrocinerea* Brot., *Salix fragilis* L., and *Salix viminalis* L. barks.

*Salix* spp.	EY (% of Dry Bark, *w*/*w*)	TPC (g GAE kg^−1^ of Dry Bark)	TPC (mg GAE g^−1^ of Extract)
*Salix atrocinerea* Brot.	15.1 ± 1.7 ^b^	44.47 ± 6.68 ^b^	293.36 ± 19.52 ^b^
*Salix fragilis* L.	9.7 ± 0.3 ^a^	17.47 ± 3.19 ^a^	179.06 ± 30.64 ^a^
*Salix viminalis* L.	10.1 ± 0.8 ^a^	24.76 ± 0.82 ^a^	246.44 ± 16.58 ^a,b^

The results represent the mean ± standard deviation. Means with different superscript minor case letters (a, b) within the same column are statistically different (one-way ANOVA, followed by Tukey’s HSD test, *p* < 0.05). GAE, gallic acid equivalents.

**Table 3:** UHPLC-DAD-MS*^n^* data of phenolic compounds detected in methanol/water/acetic acid (49.5:49.5:1) extracts of *Salix atrocinerea* Brot., *Salix fragilis* L. and *Salix viminalis* L. barks.

No.	RT (min)	Compound	λ_max_ (nm)	[M−H]^−^ (*m*/*z*)	MS*^n^* Product Ions (*m*/*z*) ^B^	Id.
1	3.79	(Epi)gallocatechin-(epi)catechin dimer isomer	233, 273	593	MS^2^: 575, 525, 467, 441, 425, 423, 407, 303, 289, 245 MS^3^: 245	[40,41]
2	6.68	B-type procyanidin dimer isomer 1	237, 277	577	MS^2^: 559, 451, 425, 407, 289, 287, 245 MS^3^: 245, 229, 205	[41]
3	6.77	Picein	229, 264	343 ^A^	MS^2^: 297, 135, 120	[11]
4	7.19	Procyanidin B1	236, 278	577	MS^2^: 559, 451, 425, 407, 289, 287, 245 MS^3^: 245	Co
5	7.37	Catechin	235, 278	289	MS^2^: 271, 245, 205, 203, 179	Co
6	7.61	B-type procyanidin dimer isomer 2	237, 278	577	MS^2^: 559, 451, 425, 407, 289, 287, 245 MS^3^: 245, 205	[41]
7	10.10	Piceol	229, 274	135	MS^2^: 93	Co
8	12.24	B-type procyanidin dimer isomer 3	241, 279	577	MS^2^: 559, 451, 425, 407, 289 MS^3^: 289, 245	[41,42]
9	12.27	Salicylic acid	241, 299	137	MS^2^: 93	[11]
10	13.86	Naringenin-*O*-hexoside isomer 1	241, 277	433	MS^2^: 433, 416, 365, 313, 271, 151 MS^3^: 151	[11]
11	14.52	Naringenin-*O*-hexoside isomer 2	241, 274	433	MS^2^: 313, 271, 251, 151 MS^3^: 151, 107	[11]
12	14.88	Quercetin 3-*O*-galactoside	241, 268, 346	463	MS^2^: 417, 395, 379, 343, 301, 300, 271, 179, 151 MS^3^: 179, 151	Co
13	18.09	Eriodictyol-*O*-hexoside isomer	238, 282, 330sh	449	MS^2^: 431, 413, 403, 381, 297; 287, 269, 175, 151, 135 MS^3^: 287, 269, 151, 135, 125, 107	[33]
14	19.88	Eriodictyol	238, 284, 330sh	287	MS^2^: 287, 151, 135, 125, 107	Co
15	23.23	Naringenin	237, 279	271	MS^2^: 227, 177, 151, 119, 107	Co

^A^ Compound 3 was detected as a formate adduct ([M+HCOO]^−^ ion). ^B^ *m*/*z* underlined was subjected to MS*^n^* analysis. The numbers (No.) of phenolic compounds correspond to the chromatographic peaks assigned in Figure 1, and the proposed chemical structures illustrated in Figure 2. Co, co-injection of a commercial standard; Id., identification; RT, retention time; sh, shoulder wavelength.

**Table 4:** Abundance of phenolic compounds in the methanol/water/acetic acid extracts (49.5:49.5:1) of *Salix atrocinerea* Brot., *Salix fragilis* L., and *Salix viminalis* L. barks.

No.	Compound	λ (nm)	mg kg^−1^ of Dry Weight			mg g^−1^ of Extract		
			*Salix atrocinerea* Brot.	*Salix fragilis* L.	*Salix viminalis* L.	*Salix atrocinerea* Brot.	*Salix fragilis* L.	*Salix viminalis* L.
1	(Epi)gallocatechin-(epi)catechin dimer isomer ^A^	280	213	−	−	1.40	−	−
2	B-type procyanidin dimer isomer 1 ^A^	280	−	−	19	−	−	0.19
4	Procyanidin B1^A^	280	404 ^F(4+5)^	−	159 ^F(4+5+6)^	2.70 ^F(4+5)^	−	1.55 ^F(4+5+6)^
5	Catechin ^A^	280	^F(4+5)^	146	^F(4+5+6)^	^F(4+5)^	1.51	^F(4+5+6)^
6	B-type procyanidin dimer isomer 2 ^A^	280	−	−	^F(4+5+6)^	−	−	^F(4+5+6)^
8	B-type procyanidin dimer isomer 3 ^B^	235	−	−	^F(8+9), G^	−	−	^F(8+9), G^
	**Σ Flavan-3-ols**		**617**	**146**	**178**	**4.10**	**1.51**	**1.73**
3	Picein ^C^	280	797	27	−	5.32	0.28	−
7	Piceol ^C^	280	1358	1537	−	9.10	15.87	−
	**Σ Acetophenones**		**2155**	**1564**	**−**	**14.42**	**16.15**	**−**
9	Salicylic acid ^B^	235	traces	58	200 ^F(8+9), G^	traces	0.59	2.00 ^F(8+9), G^
	**Σ Hydroxybenzoic acids**		**traces**	**58**	**200**	**traces**	**0.59**	**2.00**
10	Naringenin-*O*-hexoside isomer 1 ^D^	280	6	−	−	0.04	−	−
11	Naringenin-*O*-hexoside isomer 2 ^D^	280	13	−	−	0.09	−	−
13	Eriodictyol-*O*-hexoside isomer ^D^	280	−	−	51	−	−	0.50
14	Eriodictyol ^D^	280	−	−	52	−	−	0.51
15	Naringenin ^D^	280	44	5	−	0.30	0.05	−
	**Σ Flavanones**		**64**	**5**	**103**	**0.43**	**0.05**	**1.00**
12	Quercetin 3-*O*-galactoside ^E^	370	35	6	10	0.23	0.06	0.09
	**Σ Flavonols**		**35**	**6**	**10**	**0.23**	**0.06**	**0.09**
	**TOTAL**		**2871**	**1779**	**490**	**19.18**	**18.37**	**4.83**

The results represent the means obtained from *Salix* spp. bark extracts injected in triplicate (standard deviation less than 5%). Standard curves used for the quantification of phenolic compounds: ^A^ catechin; ^B^ *m*-hydroxybenzoic acid; ^C^ piceol; ^D^ naringenin; ^E^ quercetin. ^F^ The abundance of co-eluting compounds 4, 5, and 6 was determined at 280 nm, using the calibration curve of catechin. ^G^ The abundance of co-eluting compounds 8 and 9 was assayed at 235 nm, through the calibration curve of *m*-hydroxybenzoic acid, as the maximum absorbance was higher at 235 nm than at 280 nm.

**Table 5:** Antioxidant activity of methanol/water/acetic acid extracts (49.5:49.5:1) of *Salix atrocinerea* Brot., *Salix fragilis* L., and *Salix viminalis* L. bark, through DPPH• and ABTS•^+^ scavenging effects.

*Salix* spp. Bark Extract/Reference	DPPH• Scavenging Effect			ABTS•^+^ Scavenging Effect
	IC_50_ (µg mL^−1^)	IC_50_ (mg AAE g^−1^ of Dry Bark)	AAI	IC_50_ (µg mL^−1^)
*Salix atrocinerea* Brot.	10.98 ± 0.77 ^a,b^	54.41 ± 8.22 ^b^	5.64	5.58 ± 0.72 ^a,b^
*Salix fragilis* L.	23.62 ± 4.82 ^c^	16.79 ± 3.54 ^a^	2.62	10.24 ± 1.54 ^c^
*Salix viminalis* L.	14.06 ± 1.73 ^b^	28.63 ± 4.34 ^a^	4.40	7.82 ± 0.45 ^b,c^
Ascorbic acid	3.92 ± 0.08 ^a^	˗	-	3.37 ± 0.06 ^a^

The results represent the mean ± standard deviation (*n* = 9). Means with different superscript minor case letters (a–c) within the same column are statistically different (one-way ANOVA, followed by Tukey’s HSD test, *p* < 0.05). AAE, ascorbic acid equivalents; AAI, antioxidant activity index; IC_50_, inhibitory concentration at 50%.

## Supplementary Materials

The following are available online at https://www.mdpi.com/2076-3921/8/12/609/s1, Figure S1: UHPLC-UV chromatograms of methanol/water/acetic acid (49.5:49.5:1) extracts, from (**A**) *Salix atrocinerea* Brot. and (**B**) *Salix viminalis* L. barks, recorded at 280 nm. The peak numbers correspond to compounds **1–6**, **8–11** and **13**. The molecular absorption UV spectra of these compounds are also depicted; Figure S2: Mass fragmentation of a prodelphinidin dimer isomer, under negative ionization mode.
