# Supplementary material for: The Health-Promoting Potential of Salix spp. Bark Polar Extracts: Key Insights on Phenolic Composition and In Vitro Bioactivity and Biocompatibility
Source: Antioxidants (Basel). 2019 Nov 30;8(12):609. doi: 10.3390/antiox8120609 (PMC6943414; doi:10.3390/antiox8120609)
Supplement: Supplementary file 1 [file antioxidants-08-00609-s001.pdf]

(A)

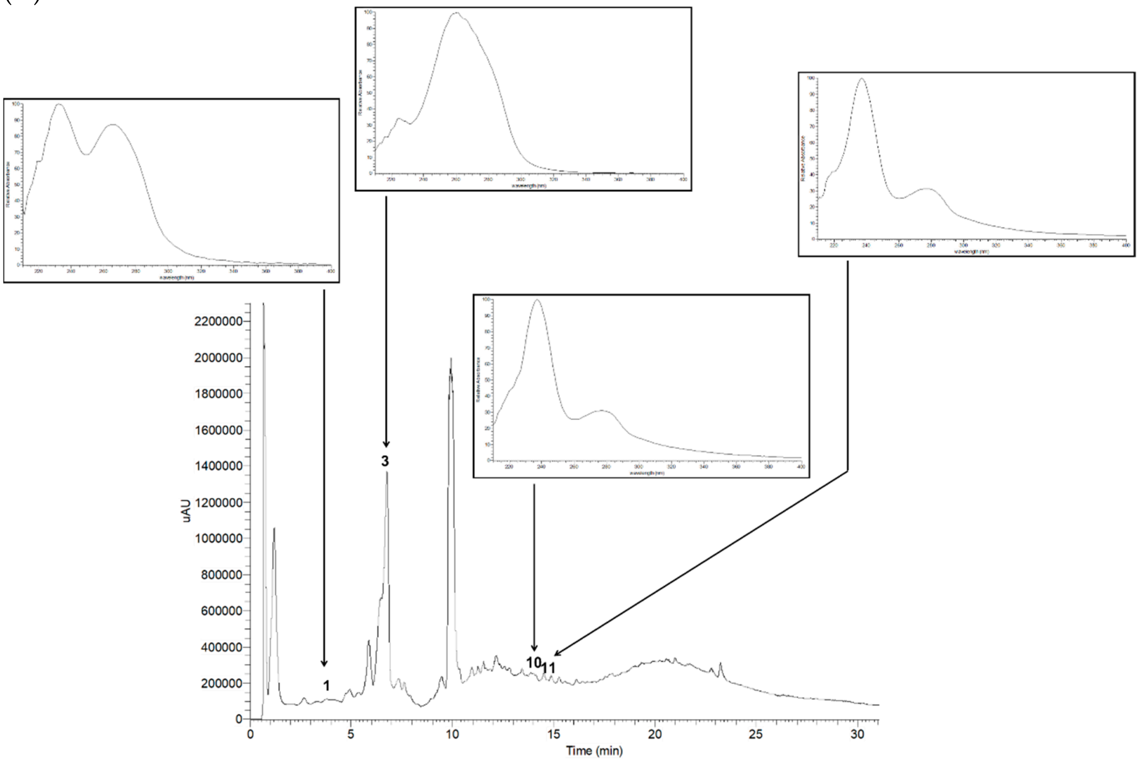

(B)

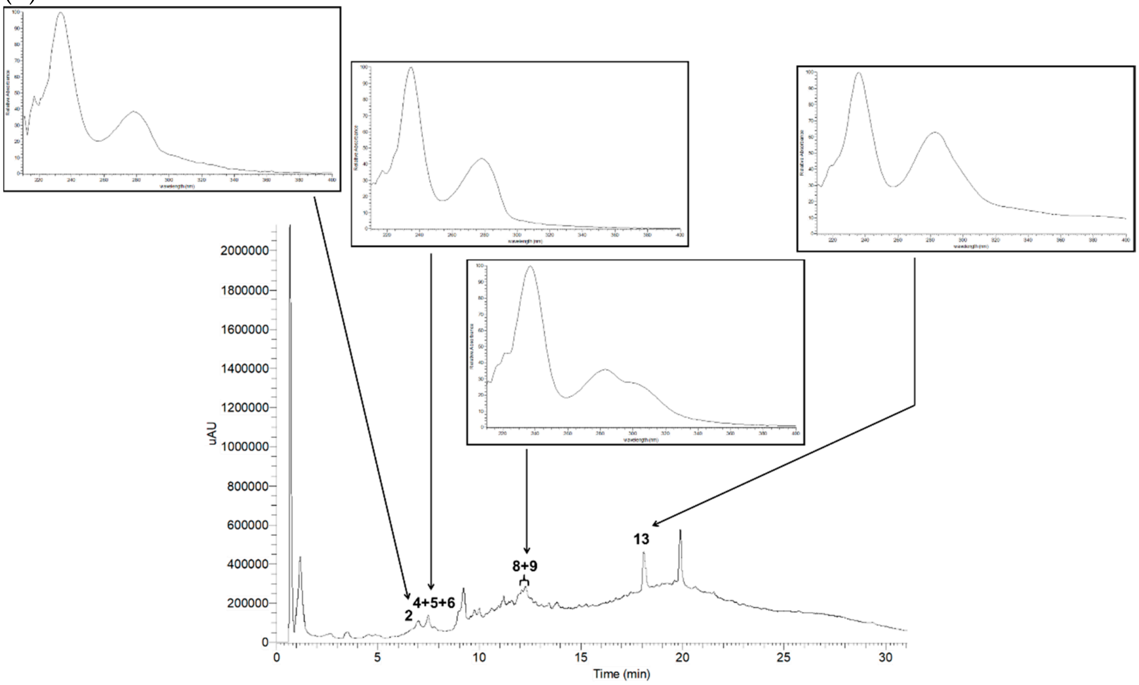

Figure S1. UHPLC-UV chromatograms of methanol/water/acetic acid (49.5:49.5:1) extracts, from (**A**) *Salix atrocinerea* Brot. and (**B**) *Salix viminalis* L. barks, recorded at 280 nm. The peak numbers correspond to compounds **1–6**, **8–11** and **13**. The molecular absorption UV spectra of these compounds are also depicted.

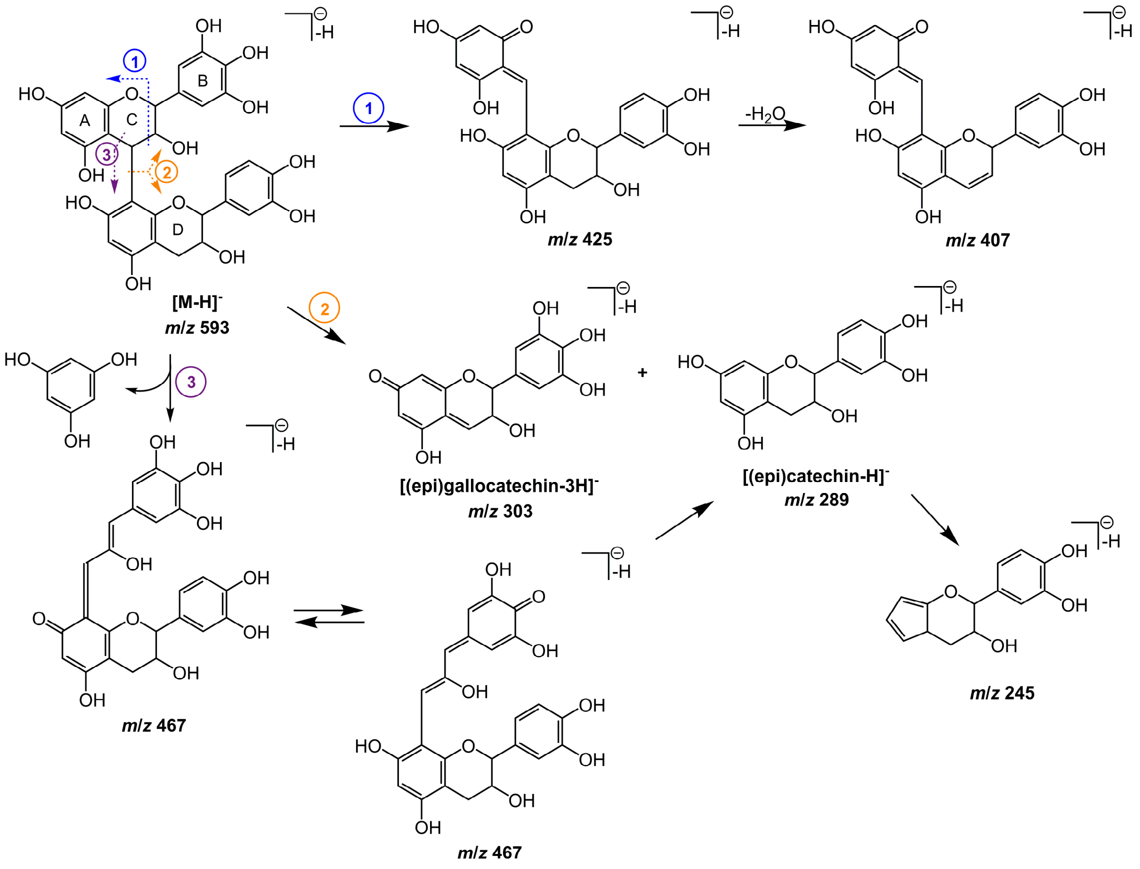

Figure S2. Mass fragmentation of a prodelphinidin dimer isomer, under negative ionization mode [1,2].

References

1. Cheynier, V. Flavonoids in wine. In *Flavonoids: chemistry, biochemistry, and applications*; Andersen, Ø.M., Markham, K.R., Eds.; CRC Press: Boca Raton, 2006; pp. 263–318.
2. Jian, Y.; Liu, R.; Chen, J.; Liu, M.; Liu, M.; Liu, B.; Yi, L.; Liu, S. Application of multifold characteristic ion filtering combined with statistical analysis for comprehensive profiling of chemical constituents in anti-renal interstitial fibrosis I decoction by ultra-high performance liquid chromatography coupled with hybrid quadrupole-orbitrap high resolution mass spectrometry. *J. Chromatog. A*, **2019**, *1600*, 197-208.
